# First investigation of *Staphylococcus argenteus* in a Brazilian collections of *S. aureus* isolated from bovine mastitis

**DOI:** 10.1186/s12917-020-02472-7

**Published:** 2020-07-20

**Authors:** Bruna F. Rossi, Érika C. R. Bonsaglia, Ivana G. Castilho, Stéfani T. A. Dantas, Hélio Langoni, José C. F. Pantoja, Ary Fernandes Júnior, Juliano L. Gonçalves, Marcos V. Santos, Rinaldo A. Mota, Vera L. M. Rall

**Affiliations:** 1 Department of Chemical and Biological Sciences, Institute of Biosciences, Sao Paulo State University - UNESP, Post Office Box 510, Botucatu, Sao Paulo 18618-970 Brazil; 2 Department of Hygiene Veterinary and Public Health, Sao Paulo State University, Botucatu, SP Brazil; 3 Department of Animal Science, School of Veterinary Medicine and Animal Science (USP), Pirassununga, SP Brazil; 4 Departament of Veterinary Medicine, Federal Rural University of Pernambuco, Recife, PE Brazil

**Keywords:** *S. aureus* complex, Staphyloxanthin, *nrps* gene, Salt egg yolk agar

## Abstract

**Background:**

*Staphylococcus argenteus* is a new specie positive coagulase staphylococci. We investigate the presence of *S. argenteus* in isolates previously classified as *S. aureus,* obtained from the milk of cows with mastitis in Brazil.

**Results:**

Among 856 *S. aureus* tested in chocolate agar, tryptone soya agar and salt egg yolk agar, white or colorless colonies were observed in 185 (21.6%) isolates. Regarding the *ctrOPQMN* operon, 111 (60%) presented the complete cluster. Despite some missing genes in this cluster, the remaining strains (74) were confirmed as *S. aureus* using the *nrps* gene.

**Conclusions:**

As far as we know, this is the first review of *S. aureus* collection in Brazil and *S. argenteus* does not appear to be a significant problem in Brazilian herds.

## Background

Mastitis is a common disease in dairy herds, causing large declines in profitability and harmful effects on animal welfare [1]. It can be caused by a variety of microorganisms and *Staphylococcus aureus* is one of the most important pathogens isolated from the milk of cows with subclinical mastitis [2].

In 2015 whole genome sequencing (WGS) confirmed that isolates from several clonal *S. aureus* lineages were sufficiently divergent to be designated as separate coagulase-positive species, included in *S. aureus* complex (SAC). While *S. schweitzeri* appears to be predominantly associated with wildlife, being restricted to Sub-Saharan Africa [3], *S. argenteus* has been reported worldwide [4–10] and is predominantly human-associated, although their recovery from several animals already occurred [3]. This new species had already been described before *S. aureus* like CC75/ST 75 [11], with an evolutionary sequence very distant from other *S. aureus* strains. Nonetheless, *S. argenteus* presents more than 60 CC, such as CC2196 (35 STs), followed by CC1594 (12 STs), CC2198 (7 STs), CC75 (5 STs), and CC2793 (2 STs) [6, 12, 13].

*S. argenteus* has an average nucleotide identity of 87.4%, corresponding to a DNA-DNA hybridization value of 33.5% compared to *S. aureus* [12, 14]. *S. argenteus* shares most of the major virulence factors of *S. aureus*, such as staphylococcal enterotoxins (SEs) [15] Antimicrobial resistance genes such as *mec*A have also been found in *S. argenteus* [16, 17] indicating the presence of mobile genetic islands and horizontal gene transition.

Phenotypic differences could be observed after growth in chocolate agar, **tryptone soya agar** (TSA) and salt egg yolk agar (SEY), as only *S. aureus* has a carotenoid pigment named staphyloxanthin [7, 12, 18] The operon *crtOPQMN* is responsible for coding the enzymes CrtO, CrtP, CrtQ, CrtM and CrtN, which are essential for staphyloxanthin synthesis [19] and the failure of any of these enzymes leads to the production of white colonies by *S. argenteus* in most agar media [9].

Genotypically, both the specific thermonuclease gene, *nuc* and 16S rRNA genes, which are used for confirmation of the *S. aureus* species, have identical sequences compared to *S. argenteus* [6]. But there is a genetic difference that can be used as a discriminative method based on deletion of 60 amino acids in the sequence of *S. aureus* nonribosomal peptide synthetase (NRPS), which does not occur *in S. argenteus*. Zhang et al. [9] discovered this deletion by comparing open-reading frames of *S. aureus* NCTC 8325 (GenBank no. NC_007795) and *S. argenteus* MSHR 1132 T (GenBank no. NC_016941) genomes, using BLAST algorithm. These genomic comparisons confirmed the presence of the intact NRPS gene in *S. argenteus*. Otherwise, more elaborate techniques are needed to discriminate *S. argenteus* from *S. aureus,* such as multilocus sequence typing (MLST) [9, 20].

Reports of this new species and improvements in identification techniques are still recent and therefore strains previously classified as *S. aureus* may be reclassified as *S. argenteus* according to the new characteristics.

Thus, the objective of the study was to investigate the presence of *S. argenteus* in isolates previously classified as *S. aureus,* obtained from the milk of cows with clinical or subclinical mastitis in different regions of Brazil.

## Results

Among the 856 strains (S1 Table) that were seeded onto the different agars, 185 (21.6%) had colonies that were white or colorless, but definitely not yellow. Interestingly, none of the 10 isolates from the state of Rio de Janeiro presented pigmentation, as indicated in Table 1. Among 185 non-pigmented strains, 60% of isolates (*N* = 111/185) presented the complete operon (Table 1). The *crtQ* gene was the most frequent, present in 157 out of 185 isolates (84.9%) (S2 Table).

**Table 1:** Presence of the operon *crtOPQMN* genes among non-pigmented *S. aureus* strains isolated from the milk of cows with clinical and subclinical mastitis, in different states in Brazil

States (N)	Non pigmented isolates N (%)	***crt***O N (%)	***crt***P N (%)	***crt***Q N (%)	***crt***M N (%)	***Crt***N N (%)	Complete Operon N (%)
SP (615)	68 (11.1)	62 (91.2)	56 (82.4)	60 (88.2)	60 (88.2)	66 (97.1)	53 (77.9)
MG (130)	82 (63.1)	39 (47.6)	61 (74.4)	63 (76.8)	48 (58.6)	56 (68.3)	33 (40.2)
PE (57)	24 (42.1)	23 (95.8)	24 (100)	23 (95.8)	24 (100)	24 (100)	23 (95.8)
PR (44)	1 (2.3)	1 (100)	1 (100)	1 (100)	1 (100)	1 (100)	1 (100)
RJ (10)	10 (100)	1 (10)	10 (100)	10 (100)	6 (60)	9 (90)	1 (10)
Total (856)	185 (21.6)	126 (68.1)	152 (82.2)	157 (84.9)	139 (75.1)	156 (84.3)	111 (60)

*SP* São Paulo, *MG* Minas Gerais, *PE* Pernambuco, *PR* Paraná, *RJ* Rio de Janeiro

Considering all genes that make up the staphyloxanthin operon (*crtO*, *crtP*, *crtQ*, *crtM*, *crtN*), 74 out of 185 (40%) non-pigmented isolates did not present at least one of them, and *crtO* was the least frequent, according to Table 2.

**Table 2:** Genotypic profiles of operon *crtOPQMN* among non-pigmented *S. aureus* strains isolated from milk of cows with clinical or subclinical mastitis

N (%)	*Operon crtOPQMN*				
	*crt*O	*crt*P	*crt*Q	*crt*M	*crt*N
111 (60)	+	+	+	+	+
18 (9.7)	–	+	+	+	+
13 (7.0)	–	–	–	–	–
7 (3.8)	+	–	–	–	+
7 (3.8)	–	+	+	–	–
6 (3.2)	–	+	+	–	+
4 (2.2)	–	–	+	+	+
3 (1.6)	–	–	–	–	+
2 (1.1)	+	+	–	+	+
2 (1.1)	–	–	+	–	–
2 (1.1)	–	+	+	+	–
1 (0.5)	+	+	+	–	–
1 (0.5)	+	+	+	–	+
1 (0.5)	+	+	+	+	–
1 (0.5)	+	–	–	–	–
1 (0.5)	+	–	+	–	+
1 (0.5)	+	–	+	–	–
1 (0.5)	–	–	+	–	+
1 (0.5)	–	+	+	–	+
1 (0.5)	–	+	–	+	+
1 (0.5)	–	+	–	–	–
185 (100)	126 (68.1)	152 (82.2)	157 (84.9)	139 (75.1)	156 (84.3)

The *nrps* gene was searched only in strains lacking at least one of the staphyloxanthin operon genes. All 74 strains presented amplicons of approximately 160 bp, considered to be *S. aureus* according to Zhang et al. [9] as *S. aureus* has a deletion in this gene while *S. argenteus* has a product of approximately 340 bp. As all strains were confirmed to be *S. aureus*, no MLST was required.

## Discussion

*S. argenteus* has been found in other regions of the world in human and animal isolates [3, 9, 21]. However, to date, this microorganism has not been reported in Brazil. According to Moradigaravand et al. [22], *S. argenteus* presents genes associated with plasmids and other mobile genetic elements previously observed with livestock-associated *S. aureus*, suggesting the need for further research into the presence of *S. argenteus* in dairy herds.

Among the 856 *S. aureus* this study, none were reclassified as *S. argenteus*, indicating the absence or low incidence of this new species in Brazilian herds. The Amazon is considered one of three main hot spots in the world, along with Australia and Southeast Asia [3] so it is important to research this new species in Brazil since the presence of a hot spot facilitates the spread of this bacteria.

Until now, few collections of *S. aureus* strains were retrospectively analyzed for the study of *S. argenteus* using culture media such as TSA [9] or chocolate agar [7]. SEY agar had been used only in isolation procedures for visualization of white colonies [18]. As a screening method, this phenotypic test, does not seem to be appropriate as we tested 856 isolates of bovine mastitis milk, with 185 (21.6%) non-pigmented and none of them were *S. argenteus*. Zhang et al. [9] tested 839 *S. aureus* isolated from food products, healthy humans and hospital infections in TSA, and 89 (10.6%) had white colonies, but only six of them (6.7%) were confirmed to be *S. argenteus*. Arguidin et al. [7] tested 1903 strains of *S. aureus* isolated from human infections in chocolate agar and found 73 (3.8%) non-pigmented isolates, but only three (4%) were *S. argenteus*.

Most (60%) of the non-pigmented *S. aureus* isolates from this study presented all operon crtOPQMN genes as did those in Zhang et al. [9] who detected the complete *crtOPQMN* operon in all their non-pigmented *S. aureus*. In those cases, the lack of pigmentation did not occur due to the absence of one of the genes so other factors must be involved in its expression or regulation.

Kitagawa et al. [23] investigated the absence of the *crtM* gene to confirm *S. argenteus* species but, as observed in this study, 46 strains confirmed as *S. aureus* also did not present the *crtM* gene, indicating that its absence is not an exclusive feature of *S. argenteus*.

Using the *nrps* gene [9], all non-pigmented isolates were confirmed as *S. aureus,* despite the absence of at least one gene in the staphyloxanthin operon. In this technique, the *nrps* gene product for *S. argenteus* has a molecular weight of approximately 340 bp while for *S. aureus* the molecular weight is reduced to approximately 160 bp. With this, the initial separation between the two species become quick and effective.

The results showed that screening tests using the lack of pigmentation in different culture media and the absence of the *crtM* gene did not show high discriminatory power in the differentiation between *S. aureus* and *S. argenteus*. On the other hand, screening using the *nrps* gene was efficient. MLST is considered the most reliable technique for confirming *S. argenteus* [9]. However, as it is a laborious technique, it needs more effective screening.

## Conclusions

As far as we know, this is the first review of an *S. aureus* collection isolated from clinical and subclinical mastitis in Brazil, and *S. argenteus* is not yet a significant problem to be considered in cases of mastitis. Besides, the development of a better phenotypic screening test is needed before conducting molecular confirmation, which is more laborious and expensive.

## Methods

### Bacterial samples

A total of 856 strains previously identified as *S. aureus* were reevaluated. All strains were isolated from the milk of cows with clinical or subclinical mastitis, from 2010 to 2019, from five Brazilian states: São Paulo (*n* = 615), Minas Gerais (*n* = 130), Pernambuco (*n* = 57), Paraná (*n* = 44) and Rio de Janeiro (*n* = 10). The strains are part of the bacterial collection found in the Food Microbiology Laboratory of the Department of Microbiology and Immunology at the Institute of Biosciences of Paulista State University in Botucatu, São Paulo (Brazil). Previously, screening tests for *S. aureus* identification were gram stain, catalase and coagulase tests, and tests for clumping factor. Confirmation through molecular typing was performed by polymerase chain reaction (PCR) using the specific nuclease (*nuc*) gene [24]. This study was approved by the Committee on Ethics in Animal Use of UNESP (0136/2017).

### Phenotypic and genotypic identification of *S. argenteus*

The initial screening was performed using SEY agar [18], chocolate agar (Oxoid, Basingstoke, UK) [7] and TSA agar (Oxoid, Basingstoke, UK) [9]. All isolates were incubated in Brain and Heart Infusion Broth (Oxoid, Basingstoke, UK) at 37 °C/24 h. After this period, an aliquot of each strain was seeded onto the different agars and incubated for 48 h at 37 °C. After this period, strains showing white colonies or without pigmentation (gray) were suspected of *S. argenteus* and selected for molecular analysis. The operon *crtOPQMN* genes (*crtO*, *crtP*, *crtQ*, *crtM* and *crtN*) were screened by PCR, according to Zhang et al. [9] in all nonpigmented isolates. The *nrps* gene [9] was tested only in isolates that did not have at least one of the *crtOPQMN* operon genes. *S. aureus* MW2, *S. aureus* WB49, *S. aureus* N315 and *S. aureus* COL were used as positive controls for the reactions.

## Supplementary information

**Additional file 1: S1 Table.** Distribuition of the non-pigmented *S. aureus* strains isolated from the milk of cows with clinical and subclinical mastitis, among the different states in Brazil.

**Additional file 2: S2 Table.** Presence of *mecA* and the operon *crt*OPQMN genes among non-pigmented S. aureus strains isolated from cows with clinical and subclinical mastitis, in different states in Brazil.

## Data Availability

All datasets generated for this study are included in the article/Supplementary Material.

## Abbreviations

WGS: Whole genome sequencing
SAC: *S. aureus* complex
SEs: Staphylococcal enterotoxins
TSA: **Tryptone Soya Agar**
SEY: Salt Egg Yolk Agar
NRPS: Nonribosomal Peptide Synthetase
MLST: Multilocus Sequence Typing
